# Performance Advantages of Left-Handed Cricket Batting Talent

**DOI:** 10.3389/fpsyg.2020.01654

**Published:** 2020-08-06

**Authors:** Jonathan D. Connor, David L. Mann, Miguel-Angel Gomez, Anthony S. Leicht, Kenji Doma

**Affiliations:** ^1^Sport and Exercise Science, James Cook University, Townsville, QLD, Australia; ^2^Department of Human Movement Sciences, Vrije Universiteit Amsterdam, Amsterdam, Netherlands; ^3^Faculty of Physical Activity and Sports Sciences, Polytechnic University of Madrid, Madrid, Spain

**Keywords:** handedness, laterality, frequency dependence, talent identification and development, talent selection

## Abstract

The purpose of this study was to examine performance advantages associated with batting stance, in the form of left- vs. right-handed dominant stance, and orthodox vs. reverse stance, of talented junior cricket batters within age-restricted competitions. Data were sourced from the national male younger age competition (YAC; Under-17; *n* = 237) and older age competition (OAC; Under-19; *n* = 302), as well as female YAC (Under-15; *n* = 234) and OAC (Under-18; *n* = 260) over a 4-year period. Left-hand dominant (LHD) batters were consistently overrepresented in the male YAC (Right: 69.2%; Left: 30.8%) and OAC (Right: 68.2%; Left: 31.8%) compared with the expected general population distribution. Male LHD batters exhibited a significantly (*p* < 0.05) higher batting aggregate (YAC: 116.82 ± 84.75 vs. 137.84 ± 89.74; OAC: 117.07 ± 89.00 vs. 146.28 ± 95.99), scored more runs (YAC: 19.65 ± 12.32 vs. 23.96 ± 14.71; OAC: 19.27 ± 12.61 vs. 23.98 ± 14.15), spent more time batting (YAC: 45.33 ± 25.89 min vs. 54.59 ± 28.62 min; OAC: 39.80 ± 21.79 min vs. 49.33 ± 27.41 min), and scored more boundary-4s per game (YAC: 1.83 ± 1.40 vs. 2.44 ± 1.87; OAC: 1.76 ± 1.32 vs. 2.19 ± 1.83), across both YAC and OAC groups with small effect sizes. No overrepresentation was present for either female group (YAC, Right: 88.5%/Left: 11.5%; OAC, Right: 90.0%/Left: 10.0%). Female LHD batters exhibited significantly higher batting aggregate (68.97 ± 53.17 vs. 102.96 ± 73.48), batting average (13.24 ± 10.88 vs. 17.75 ± 12.28), and spent more time batting per game (25.52 ± 15.08 vs. 37.75 ± 26.76 min), but only at the OAC level with small-moderate effects sizes. Finally, there were few performance advantages identified to batting with a reverse stance, with further work needed to clarify any potential biomechanical benefits. Team selection practices may exploit the left-handed advantage by over-selecting talented left-handed junior cricketers. Practical implications for coaches include creating practice environments that negate the negative frequency-dependent selection, such as providing more practice opportunities for their players against left-handed opponents.

## Introduction

The overrepresentation of left-handers, compared with the expected distribution seen in the general population, has been well established within interactive time-restrictive skills (Hagemann, 2009; Harris, 2010; Loffing, 2017). The most prominent explanation for the left-hander’s advantage has been the *negative frequency-dependent hypothesis* (Raymond et al., 1996; Grouios, 2004). This hypothesis presumes that athletes in interactive sports are much more likely to play and practice against right-handed opponents. As a result, these athletes develop both greater familiarity and highly specific skills to anticipate the action outcomes of their right-handed opponents via attunement to crucial perceptual information (Loffing and Hagemann, 2014). While a greater proportion of left-handers are commonly observed in high-level sports (∼30%) compared to the general population incidence (∼10–15%), a specific left-handed performance advantage during competition has not been clearly reported (Grouios et al., 2000; Loffing, 2017).

Evidence of a relative advantage for anticipating the actions of a right-handed (or footed) opponent has been reported across a number of interactive tasks including predicting left- and right-handed serves in volleyball (Loffing et al., 2012b), predicting kicking direction from left- and right-footed opponents in soccer (McMorris and Colenso, 1996) and predicting left- and right-handed serves in tennis (Loffing et al., 2009). Loffing et al. (2012a) concluded that the performance advantage for left-handers may be attributed to more highly skilled visual perception, stemming from more frequent encounters with right-handers (Loffing et al., 2012b). Unsurprisingly, handedness has also been reported as a potentially influential factor in team selection, with Brooks et al. (2004) finding that the most successful teams at the 2003 Cricket World Cup had nearly 50% representation of left-handed cricket batsmen.

Additional explanations for the prominence of left-handers within elite sporting teams have also included potential advantages afforded by sport-specific rule constraints and innate technical advantages (Grondin et al., 1999). For instance, Raymond et al. (1996) suggested that a greater frequency of left-handers may result from the constraints (e.g., rules) within the performance environment. For example, baseball batters who bat left-handed may have tactical or strategic advantages in that (i) their stance places them closer to first base toward which they must run and (ii) their momentum at the end of the swing is already directed toward first base (as opposed to toward third base for batters with a right-handed stance). Similarly, the leg-before-wicket rule in cricket may advantage cricket batters who bat left-handed. In the familiar situation of facing a right-handed bowler, it is much more challenging for a left-handed batter to be dismissed leg-before-wicket due to particularities in where the ball must bounce to be adjudicated as “out.” However, potential advantages of handedness within interactive sporting tasks remain in development (Mann et al., 2017). Mann et al. (2016) examined the prevalence of left-handedness in elite cricket batters and found not only an increased frequency of left-hand dominant (LHD) stance cricket batters, but also a tendency for batters (particularly left-handers) to adopt a “back-to-front” batting stance. The reverse stance hypothesis was therefore proposed as a potential technical advantage when holding a cricket bat with the dominant hand positioned further away from the hitting end of the bat (Mann et al., 2016). Interestingly, this stance was more common in LHD stance batters and it is unknown as to what degree either factor influences performance.

While there is evidence that left-handedness—and perhaps a “reversed stance”—may be advantageous at the elite level of competition (Mann et al., 2016), much less is known about the age, gender, or performance level at which these advantages might emerge, particularly as other talent selection bias has been previously highlighted in cricket (Connor et al., 2019). If the *negative frequency-dependent hypothesis* were apparent, then it might be reasonable to expect the advantage of the left-handed cricket batters to increase over time due to a progressive accrual of experience competing against right vs. left-handed opponents. Loffing et al. (2012a) reported a moderate positive impact of left-handedness on tennis performance, which decreased over a time span of years for males, and was almost entirely absent in professional female tennis players. These results suggest that a left-hander’s advantage may primarily happen within lower performance levels and be a rarity at the elite level. Alternately, others have shown that the magnitude of some performance advantages increases and can become increasingly more important, along the developmental pathway (Weissensteiner et al., 2008). Therefore, development of a handedness advantage at the junior level may be important for future performance, yet remains to be confirmed.

The aim of this study was to examine the magnitude of performance advantages associated with batting stance, in the form of left- vs. right-handed dominant stance, and orthodox vs. reverse stance, in junior cricket players within age-restricted competitions. Both male and female players were examined to account for any potential sex differences in performance due to handedness (Loffing, 2017). We hypothesized that the magnitude of the performance advantage, associated with batting handedness (left vs. right) and batting stance (orthodox vs. reverse), would be larger for both male and female cricket batters participating in the older age competition (OAC).

## Materials and Methods

### Participants and Procedures

Playing data for all male younger-age cricketers who competed at the Annual National Junior championships in Australia between 2012 and 2015 were obtained from an open source online database^1^ (excluding male Under-17, which included data from 2013 to 2015) and confirmed via the National Sporting body’s database. Players were then separated into whether they competed in the younger age competition (YAC; male Under-17 and female Under-15) or the OAC (male Under-19 and female Under-18). Batting handedness was determined by the players throwing hand, in line with previous work (Cairney et al., 2018). Players were included and categorized as batters if they (1) were listed by the coach as a batter in the top seven positions and (2) batted in these positions during at least three innings of the tournament. In total, 237 YAC and 302 OAC-19 males, and 234 YAC and 260 OAC female players met the criteria. This research received institutional approval from James Cook University (H6267) and was conducted in accordance with the National Statement on Ethical Conduct in Human Research (2007).

### Data Analysis

Measures of batting performance were collated from the tournament database. Two measures of absolute performance were recorded: (i) *batting aggregate*, representing the total number of runs scored at the tournament, and (ii) *number of innings batted*. Relative measures included (i) *batting average* (i.e., number of runs scored divided by number of dismissals), (ii) *runs per innings* (i.e., number of runs scored divided by number of innings), (iii) *strike rate* (i.e., runs scored per 100 balls), (iv) *boundary-4s per game* (i.e., average number of “fours” scored per innings), (v) *boundary-6s per game* (i.e., average number of “sixes” scored per innings), and (vi) *time per innings* (i.e., average time spent batting per innings). Finally, the percentage of innings was recorded where the batter was (i) *not dismissed* (i.e., not outs), and where the batter had scored (ii) *ducks* (i.e., scores of 0 runs per player) and (iii) *centuries* (i.e., scores of 100 runs or more per player). Participant’s batting handedness was recorded as either right-hand dominant (RHD) or LHD. Batting stance was also recorded as being *orthodox* (dominant hand placed on the bottom of the bat) if the dominant hand matched the batting handedness (e.g., left-handed batter, LHD) or *reversed* (dominant hand placed on top off the bat) if the dominant hand did not match the batting handedness (e.g., right-handed batter, LHD).

### Statistical Analysis

All data were analyzed using the Statistical Package of Social Sciences (SPSS, version 25, IBM Corp., Armonk, NY, United States) with the alpha level set at ≤ 0.05 and descriptive information expressed as mean ± standard deviation. First, any over- or underrepresentation of left-handedness, when compared with the proportions expected from the general population (RH = 85%; LH = 15%; Raymond et al., 1996; Johnston et al., 2009; Medland et al., 2009), was determined using chi-squared goodness-of-fit tests. Measures of batting performance measures were compared between groups (e.g., left- and right-handed stances; orthodox and reverse stances) using separate one-way MANOVAs for each group. Effect sizes (95% confidence interval) were calculated using Cohen’s *d*, and reported as 0.2, 0.5, and 0.8 as small, moderate, and large, respectively (Cohen, 1988).

## Results

### Representation

There was a significant overrepresentation of LHD cricket batters compared with the general population at both the YAC and OAC male level (Table 1). For females, there was no significant overrepresentation of LHD cricket batters at either the YAC or OAC level when compared to the left-handedness expected of the general population (Table 2).

**TABLE 1:** Number (and percentage) of left- and right-hand dominant male and female batters.

	Competition level	Right-handed batting stance	Left-handed batting stance	χ^2^	*p*-Value
Male	YAC	164 (69.2%)	73 (30.8%)	19.58	<0.001
	OAC	206 (68.2%)	96 (31.8%)	22.14	<0.001
Female	YAC	207 (88.5%)	27 (11.5%)	0.96	0.33
	OAC	234 (90.0%)	26 (10.0%)	1.96	0.16

*General population left-handedness = 15%.*

**TABLE 2:** Mean (±standard deviation) batting performances measures for left- and right-handed male batters during the YAC and OAC and effect size calculations between batters with associated 95% confidence interval (CI).

		Right-handed batting stance	Left-handed batting stance	Effect size (95% CI)	*p*-Value
Matches	YAC	6.26 ± 1.56	5.99 ± 1.58	0.17 (−0.11,0.45)	0.22
	OAC	5.80 ± 1.43	5.83 ± 1.63	0.02 (−0.26,0.22)	0.86
Innings	YAC	5.65 ± 1.63	5.85 ± 1.51	0.12 (−0.40,0.16)	0.39
	OAC	5.77 ± 1.53	5.71 ± 1.46	0.04 (−0.20,0.28)	0.74
Batting aggregate	YAC	116.82 ± 84.75	137.84 ± 89.74	0.24 (−0.49,0.00)	< 0.05
	OAC	117.07 ± 89.00	146.28 ± 95.99	0.32 (−0.60,−0.04)	< 0.05
Batting average	YAC	22.78 ± 14.41	27.63 ± 17.09	0.32 (−0.56,−0.07)	< 0.05
	OAC	23.76 ± 17.02	28.19 ± 17.62	0.26 (−0.54,0.02)	0.07
Runs per innings	YAC	19.65 ± 12.32	23.96 ± 14.71	0.33 (−0.57,−0.08)	< 0.01
	OAC	19.27 ± 12.61	23.98 ± 14.15	0.36 (−0.64,−0.08)	< 0.05
Batting time (per game)	YAC	45.33 ± 25.89	54.59 ± 28.62	0.35 (−0.59,−0.10)	< 0.01
	OAC	39.80 ± 21.79	49.33 ± 27.41	0.40 (−0.68,−0.12)	< 0.01
Boundary-4s (per game)	YAC	1.83 ± 1.40	2.44 ± 1.87	0.39 (−0.64,−0.15)	< 0.01
	OAC	1.76 ± 1.32	2.19 ± 1.83	0.29 (−0.56,0.00)	< 0.05
Boundary-6s (per game)	YAC	0.24 ± 0.38	0.26 ± 0.38	0.05 (−0.29,0.19)	0.69
	OAC	0.21 ± 0.35	0.26 ± 0.35	0.17 (−0.44,0.11)	0.25
Strike rate	YAC	57.11 ± 21.84	58.86 ± 18.25	0.08 (−0.33,0.16)	0.50
	OAC	59.43 ± 22.68	60.75 ± 20.67	0.06 (−0.34,0.22)	0.67
Non-dismissal (%)	YAC	12.07 ± 15.54	8.87 ± 12.99	0.09 (−0.15,0.33)	0.47
	OAC	15.45 ± 18.59	13.72 ± 15.35	0.10 (−0.18,0.38)	0.29
Ducks scored	YAC	11.58 ± 11.37	7.25 ± 13.21	0.34 (0.06,0.62)	< 0.05
	OAC	10.31 ± 14.21	7.521 ± 1.41	0.21 (−0.04,0.45)	0.09
Centuries scored	YAC	1.88 ± 8.07	3.18 ± 5.90	0.20 (−0.47,0.08)	0.17
	OAC	1.29 ± 4.71	3.29 ± 7.57	0.35 (−0.59,−0.10)	< 0.05

### Batting Performance—Males

Irrespective of their age group, when compared to RHD cricket batters, LHD male batters had significantly higher batting aggregates, runs per innings, time spent batting, and boundary-4s (small ES, Table 2). LHD in the YAC group had a significantly higher batting average (*p* < 0.05), whereas the advantage in the OAC group for LHD batters was marginal (*p* = 0.07). LHD batters also exhibited significantly less ducks in the YAC group only, and significantly more centuries scored in the OAC group (*p* < 0.05). All other variables across both YAC and OAC groups were similar, including the number of matches, innings, boundary-6s, and strike rate, with all exhibiting a small ES (Table 2).

### Batting Performance—Females

There was no significant difference between LHD and RHD batters for each performance measure at the YAC level (all small ES, Table 3). At the OAC level, LHD exhibited significantly greater batting aggregate, batting average, average runs per game, and batting time (*p* < 0.05; small-moderate ES, Table 3). No other differences were observed between LHD and RHD batters at the OAC level for number of matches or innings, boundary-4s and -6s scored, strike rate, non-dismissals, and ducks or centuries scored (small ES, Table 3).

**TABLE 3:** Mean (±standard deviation) batting performances measures for left- and right-handed female batters during the YAC and OAC and effect size calculations between batters with associated 95% confidence interval (CI).

		Right-handed batting stance	Left-handed batting stance	Effect size (95% CI)	*p*-Value
Matches	YAC	7.54 ± 0.51	7.40 ± 0.50	0.28 (−0.13,0.69)	0.18
	OAC	7.50 ± 0.65	7.56 ± 0.58	0.08 (−0.48,0.32)	0.69
Innings	YAC	6.46 ± 1.65	6.60 ± 1.32	0.09 (−0.50,0.32)	0.67
	OAC	6.22 ± 1.32	6.67 ± 1.41	0.34 (−0.74,0.07)	0.10
Batting aggregate	YAC	76.96 ± 63.48	92.24 ± 63.02	0.24 (−0.65,0.17)	0.25
	OAC	68.97 ± 53.17	102.96 ± 73.48	0.61 (−1.01,−0.20)	< 0.01
Batting average	YAC	15.01 ± 13.03	17.00 ± 12.15	0.15 (−0.57,0.26)	0.47
	OAC	13.24 ± 10.88	17.75 ± 12.28	0.41 (−0.81,0.00)	< 0.05
Runs per innings	YAC	11.25 ± 8.23	13.57 ± 8.09	0.28 (−0.69,0.13)	0.18
	OAC	10.66 ± 7.84	14.54 ± 8.89	0.49 (−0.89,−0.08)	< 0.05
Batting time	YAC	21.64 ± 12.50	24.76 ± 12.90	0.25 (−0.66,0.17)	0.24
	OAC	25.52 ± 15.08	37.75 ± 26.76	0.73 (−1.13,−0.32)	< 0.01
Boundary-4s	YAC	1.22 ± 1.08	1.43 ± 1.08	0.20 (−0.61,0.22)	0.35
	OAC	0.74 ± 0.75	1.00 ± 1.02	0.34 (−0.74,0.07)	0.10
Boundary-6s	YAC	0.03 ± 0.11	0.02 ± 0.07	0.03 (−0.39,0.44)	0.90
	OAC	0.02 ± 0.06	0.00 ± 0.03	0.21 (−0.20,0.61)	0.31
Strike rate	YAC	56.61 ± 23.60	63.33 ± 22.34	0.29 (−0.70,0.13)	0.18
	OAC	50.19 ± 20.33	50.75 ± 12.91	0.03 (−0.43,0.37)	0.89
Non-dismissals (%)	YAC	19.28 ± 17.80	17.44 ± 16.72	0.10 (−0.31,0.52)	0.62
	OAC	16.43 ± 15.82	15.22 ± 12.54	0.08 (−0.32,0.48)	0.70
Ducks scored	YAC	0.15 ± 0.17	0.16 ± 0.14	0.06 (−0.46,0.34)	0.68
	OAC	0.14 ± 0.14	0.14 ± 0.16	0.00 (−0.41,0.41)	0.98
Centuries scored	YAC	0.00 ± 0.02	0.00 ± 0.00	0.00 (−0.40,0.40)	0.47
	OAC	0.00 ± 0.03	0.00 ± 0.00	0.00 (−0.41,0.41)	0.63

### Batting Stance

Younger age competition reverse stance male cricket batters had significantly greater batting time (small-moderate ES) compared with orthodox stance batters (*p* < 0.05) while no other significant differences between batting variables were evident between the different stances (trivial to small-moderate ES, Table 4). The OAC reverse stance batters also had significantly greater batting time as well as greater boundary-4s (small-moderate ES, Table 4) compared with orthodox stance batters (*p* < 0.05). There were no other significant differences evident between the different stances for OAC level (trivial to small-moderate ES, Table 4).

**TABLE 4:** Mean (±standard deviation) batting performances measures for orthodox and reverse stance during the male YAC and OAC and effect size calculations between batters with associated 95% confidence interval (CI).

		Orthodox batting stance	Reverse batting stance	Effect size (95% CI)	*p*-Value
Matches	YAC	6.21 ± 1.55	6.04 ± 1.62	0.11 (−0.21,0.42)	0.50
	OAC	5.83 ± 1.44	5.75 ± 1.64	0.05 (−0.21,0.31)	0.71
Innings	YAC	5.69 ± 1.59	5.73 ± 1.62	0.03 (−0.34,0.29)	0.85
	OAC	5.82 ± 1.52	5.53 ± 1.43	0.19 (−0.59,−0.06)	0.16
Batting aggregate	YAC	121.44 ± 90.45	142.90 ± 96.54	−0.23 (−0.55,0.08)	0.15
	OAC	120.99 ± 86.07	131.45 ± 89.08	−0.12 (−0.38,0.14)	0.37
Batting average	YAC	24.71 ± 17.50	26.59 ± 16.51	−0.11 (−0.42,0.21)	0.50
	OAC	23.83 ± 15.03	25.89 ± 16.71	−0.13 (−0.40,0.13)	0.32
Runs per innings	YAC	19.97 ± 13.13	23.45 ± 13.43	−0.26 (−0.58,0.05)	0.10
	OAC	20.29 ± 12.42	23.31 ± 15.46	−0.23 (−0.49,0.04)	0.09
Batting time (per game)	YAC	41.14 ± 23.53	48.58 ± 25.00	−0.31 (−0.63,0.00)	< 0.05
	OAC	46.45 ± 25.73	54.02 ± 30.43	−0.28 (−0.54,−0.02)	< 0.05
Boundary-4s (per game)	YAC	1.84 ± 1.53	2.07 ± 1.39	−0.15 (−0.47,0.16)	0.35
	OAC	1.90 ± 1.42	2.41 ± 1.99	−0.32 (−0.59,−0.06)	< 0.05
Boundary-6s (per game)	YAC	0.21 ± 0.34	0.29 ± 0.38	−0.23 (−0.54,0.09)	0.16
	OAC	0.24 ± 0.38	0.26 ± 0.41	−0.04 (−0.30,0.23)	0.78
Strike rate	YAC	59.48 ± 22.46	61.13 ± 20.61	−0.07 (−0.39,0.24)	0.64
	OAC	57.73 ± 21.06	57.48 ± 19.89	0.01 (−0.25,0.28)	0.93
Non-dismissal (%)	YAC	16.02 ± 18.34	10.78 ± 14.21	0.30 (−0.02,0.61)	0.06
	OAC	12.71 ± 15.54	8.90 ± 13.17	0.25 (−0.01,0.52)	0.06
Ducks scored	YAC	0.11 ± 0.13	0.08 ± 0.10	0.21 (−0.11,0.52)	0.20
	OAC	0.10 ± 0.14	0.07 ± 0.11	0.24 (−0.02,0.51)	0.07
Centuries scored	YAC	0.02 ± 0.06	0.03 ± 0.08	0.15 (−0.46,0.17)	0.36
	OAC	0.02 ± 0.05	0.03 ± 0.07	0.22 (−0.49,0.04)	0.10

Female cricket batters with a reverse batting stance in the YAC group exhibited significantly greater batting aggregate and batting time (*p* < 0.05; small ES, Table 5). Batting average was also greater for reverse stance female batters (*p* < 0.05). No other differences were evident between reverse and orthodox stance for the YAC group. There were also no significant differences between stances for any performance measure at the OAC level (trivial to small-moderate ES, Table 5).

**TABLE 5:** Mean (±standard deviation) batting performance measures for orthodox and reverse stance during the female YAC and OAC tournaments and effect size calculations between batters with associated 95% confidence interval (CI).

		Orthodox batting stance	Reverse batting stance	Effect size (95% CI)	*p*-Value
Matches	YAC	7.55 ± 0.51	7.36 ± 0.49	0.37 (−0.04,0.78)	0.08
	OAC	7.49 ± 0.66	7.60 ± 0.55	−0.17 (−0.53,0.19)	0.36
Innings	YAC	6.44 ± 1.64	6.80 ± 1.29	−0.23 (−0.64,0.19)	0.28
	OAC	6.25 ± 1.31	6.37 ± 1.50	−0.09 (−0.45,0.27)	0.62
Batting aggregate	YAC	75.79 ± 62.60	103.08 ± 67.59	−0.43 (−0.85,−0.02)	< 0.05
	OAC	72.24 ± 57.47	76.60 ± 53.07	−0.08 (−0.44,0.28)	0.68
Batting average	YAC	14.82 ± 13.00	18.78 ± 12.02	−0.45 (−0.72,0.11)	< 0.05
	OAC	13.83 ± 11.58	13.38 ± 8.10	0.04 (−0.32,0.40)	0.83
Runs per innings	YAC	11.11 ± 8.18	14.82 ± 8.09	−0.31 (−0.87,−0.04)	0.15
	OAC	11.09 ± 8.29	11.17 ± 6.58	−0.01 (−0.37,0.35)	0.96
Batting time (per game)	YAC	21.38 ± 12.48	27.15 ± 12.22	−0.46 (−0.88,−0.05)	< 0.05
	OAC	26.99 ± 16.93	26.58 ± 19.02	0.02 (−0.34,0.38)	0.90
Boundary-4s (per game)	YAC	1.21 ± 1.07	1.55 ± 1.12	−0.31 (−0.72,0.10)	0.14
	OAC	0.77 ± 0.81	0.78 ± 0.68	−0.02 (−0.37,0.34)	0.93
Boundary-6s (per game)	YAC	0.03 ± 0.11	0.02 ± 0.07	0.08 (−0.33,0.49)	0.70
	OAC	0.02 ± 0.06	0.01 ± 0.03	−0.02 (−0.20,0.52)	0.39
Strike rate	YAC	56.66 ± 24.10	62.91 ± 16.60	−0.27 (−0.68,0.15)	0.21
	OAC	49.42 ± 18.65	54.99 ± 24.10	−0.28 (−0.64,0.08)	0.12
Non-dismissal (%)	YAC	19.27 ± 17.98	17.52 ± 14.73	0.10 (−0.31,0.51)	0.64
	OAC	16.45 ± 15.78	15.34 ± 13.72	0.07 (−0.29,0.43)	0.70
Ducks scored	YAC	0.15 ± 0.17	0.13 ± 0.12	0.12 (−0.29,0.54)	0.20
	OAC	0.14 ± 0.14	0.17 ± 0.16	0.22 (−0.58,0.14)	0.07
Centuries scored	YAC	0.00 ± 0.02	0.00 ± 0.00	0.15 (−0.26,0.57)	0.36
	OAC	0.00 ± 0.03	0.00 ± 0.00	0.10 (−0.26,0.46)	0.10

## Discussion

The purpose of this study was to examine the magnitude of performance advantages associated with various batting stances, within an interactive striking task. We found that LHD cricket batters were overrepresented in competition for junior males, but not for females. Furthermore, male LHD batters in both groups scored more runs and batted for longer periods of time when compared with their right-handed counterparts. For female LHD cricket batters, only the OAC group scored more runs and spent more time batting compared with their right-handed counterparts. Reverse stance batters exhibited some performance advantages over orthodox stance batters, including greater batting time (YAC and OAC) and boundary-4s (OAC) for males, and greater runs scored and batting time for female cricket batters (YAC). These findings demonstrate evidence of a small left-handed advantage in cricket batting, with limited performance advantage for either orthodox or reverse batting stance at the youth representative level.

Left-handed male cricket batters were found to be more prevalent in junior elite competition when compared to the proportion expected from the general population (Table 1). Similar findings have been reported at the elite level for males, whereby batting records from the 2003 cricket World Cup showed that the most successful teams in the competition had close to 50% representation of left-handed batsmen (Brooks et al., 2004). Mann et al. (2016) investigated left- and right-handedness among the greatest elite male cricket batters to have played the game, and reported comparable proportions to this study (left-handers = 33%; right-handers = 67%). Additionally, Mann et al. recorded the handedness of inexperienced cricket batters, finding levels comparable to the general population (left-handers = 15.2%; right-handers = 84.8%). Collectively, the current and prior findings confirm an overrepresentation of left-handed cricket batters at the elite junior and senior levels (Mann et al., 2016; Loffing, 2017).

Hagemann (2009), in his review of advantages according to handedness, reported that overrepresentations commonly occur in sporting tasks that involve the anticipation of an opponent’s action. There has also been further suggestion that left-handedness is particularly evident during more temporally demanding sporting tasks such as tennis, cricket batting, or baseball batting (Loffing et al., 2010, 2012b; Loffing, 2017), although the exact temporal demand threshold at which a handedness advantage becomes apparent is unclear. It is thought that the *negative frequency* effect may influence the acquisition of perceptual or strategic skills required to outperform a left-handed opponent (Schorer et al., 2012). Specifically, a greater proportion of practice time would likely be spent against right-handed opponents, which may afford better attunement to the perceptual information available from right-handers. This may result in increased difficulty when responding to or anticipating the actions of left-handers (Schorer et al., 2012), given the fewer experiences practicing against skillful left-handers (compared with right-handers).

This study also highlighted a number of performance advantages for left-handed male cricket batters, irrespective of their elite age level competition (Tables 2, 3). LHD batters had greater run scoring metrics (e.g., batting aggregate, batting average, runs per innings), batted for a longer period of time, and struck more boundary-4s. It should be noted that while there was a significant difference between these measures, there were also small effect sizes between LHD and RHD batters. No differences between strike rate, boundary-6s, and non-dismissals suggest that LHD batters do not score at a faster rate, nor are they dismissed by the opposition any less often than RHD (e.g., see Table 2). Some studies have also reported performance metric advantages for left-handers in other interceptive striking sports such as tennis and baseball (Grondin et al., 1999; Loffing et al., 2010), though there has been far less reported evidence for interactive sports which do not involve a striking implement (Baker and Schorer, 2013; Tirp et al., 2014; Cingoz et al., 2018). Rather than being explained by innate factors, the superiority of left-handers during sporting tasks can be explained due to a combination of tactical or strategic factors (Grouios et al., 2000; Loffing et al., 2012a). One of these crucial factors is the negative frequency effect of left-handers, which highlights the advantage left-handers possess due to their unfamiliarity of their competitors. Schorer et al. (2012) intervention study further supported the importance of familiarity and practice against left-handers, which reported that correctly anticipating the actions of left- or right-handers was dependent on whether participants had been practicing against a left- or right-handed opponent. The findings of these studies have important practical implications for coaches and athletes. In order to minimize the negative frequency effect of left-handers, training environments should be designed in an effort to provide greater opportunities for athletes to practice against left-handed competitors.

Interestingly, the prevalence of left-handedness in female cricket batters at the YAC and OAC level corresponded with levels expected within the general population (Table 3) with differences in performance metrics between LHD and RHD batters at the OAC level only. These findings support Loffing (2017) examination of left-handed overrepresentation in sports that have significant temporal demands, which are reportedly greater in the men’s game due to faster bowling speeds (Felton et al., 2019). The performance advantage observed for OAC LHD batters may therefore be explained due to increased temporal demands within the older age group competition compared with the younger age group competition (Pyne et al., 2006; Connor et al., 2018). One other possible explanation for the contrasting differences in left-handedness prevalence and performance advantages is the degree to which there is competition for selection. Selection pressures within the “talent system” increase as selection between athletes becomes more competitive. In their review of other selection biases (i.e., relative age effects), Musch and Grondin (2001) described how selection biases increased, as the size of the playing pool from which players were selected from also increased. The 2018/19 cricket participation census released by Cricket Australia highlighted the vast difference between the percentage of male (∼70%) and female players (∼30%) participating in cricket. Therefore, a smaller population pool from which to select players may attenuate certain biases, such as handedness, and the performance advantage they afford male cricketers. Loffing (2017) highlighted similar findings in racket sports, which reported an overrepresentation of left-handers compared to the general population in elite male competition, but not the female competition. It is currently unclear whether the reduced handedness bias is due to potentially less selection pressures or the temporal demands of the sport. Further research is required to investigate laterality in female competitions.

Finally, to better understand any potential left-handed advantage, moderating factors such as the adoption of a “reversed” stance (Mann et al., 2016) were also analyzed in the current study. The reversed-stance advantage hypothesis (Mann et al., 2016) proposed an explanation for certain performance advantages above and beyond the negative frequency-dependent effects available to all left-handers. Their findings highlighted that professional cricket batsmen were seven times more likely to adopt a reversed stance than inexperienced batsmen. The current study provided some preliminary evidence for reverse stance batters having limited performance advantage at the talent pathway level. One explanation for these small performance advantages may be due to a potential technical advantage imposed by having a dominant hand on the top of the bat handle, rather than the bottom. The role of the top hand is thought to be primarily responsible for guiding the path of the bat in order to intercept the ball (Stretch et al., 1995, 1998). Therefore, it may be more advantageous for the dominant hand to be placed on top of the bat handle (reverse stance) rather than the bottom when attempting to forcefully contact the ball. However, more empirical work is required on the reverse-stance before any practical recommendations can or should be made to coaches, practitioners, and learners.

There are some limitations to consider within this study. First, handedness was compared to the expected distribution within the general sporting population, which may not accurately reflect the exact percentage of amateur level cricketers who are left-handed. Future studies would benefit from analyzing a sample of amateur or recreational level players within the population being investigated (Mann et al., 2016). Analysis was also specific to cricket batters and may not generalize to cricket bowlers. The use of frequencies and counts for those variables in the current study should be analyzed as independent measures for predictive (e.g., counts used via linear regression) and association (e.g., frequencies used via Crosstabs Commands) models in future work. Second, cricketers in this study were categorized based on whether they competed in a younger age or older age national competition, which relies on chronological age cutoffs. Previous research has highlighted that selection in these competitions can heavily bias the sample population toward relative older players (Connor et al., 2019). Finally, the nature of this study does not allow for the analysis of explanatory factors of various batting stances. Rather, it is an examination of batting stance and its impact on real-world sporting performance. Further investigations seeking to explore the mechanisms underpinning batting stance could focus on potential biomechanical differences across various skill levels (Stretch et al., 1995; Sarpeshkar and Mann, 2011).

## Conclusion

The current study has demonstrated a small left-handed performance advantage for cricket batters at the junior national representative level. This advantage was evident for both male and female batters despite overrepresentation of left-handers being evident in the male pathway system only. Highly competitive and large talent selection pools, coupled with temporally demanding sporting tasks, are proposed to explain the increased representation of left-handers in elite junior cricket. The lower frequency of female left-handers (in this case, compared with right-handers) would mean that there are also less practice opportunities against left-handers, which may partially explain the left-hander’s performance advantage for female cricket. Further, this study provided evidence for the reverse stance hypothesis contributing a limited performance advantage to cricket batting. Together, these findings highlight the emergence of selection and performance biases occurring at the male and female youth representative level. Practical implications for coaches include creating practice environments that minimize the negative frequency effect, such as providing more practice opportunities against left-handed opponents. Future performance analysis research is encouraged to examine whether selecting a greater number of left-handers increases the likelihood of winning matches.

## Data Availability Statement

All datasets generated for this study are included in the article/Supplementary Material.

## Ethics Statement

The study was conducted according to the ethical guidelines of the authors’ affiliated institution. Written informed consent from the participants’ legal guardian/next of kin was not required to participate in this study as analyzed data were archival data available in the public domain.

## Author Contributions

JC contributed to the conception and design of the work, writing of the manuscript, and analysis and interpretation of data. DM, AL, and KD contributed to the writing and critiquing of the manuscript and interpretation of data. M-AG contributed to the critiquing of the manuscript, data analysis, and interpretation of the data. All authors contributed to the article and approved the submitted version.

## Conflict of Interest

The authors declare that the research was conducted in the absence of any commercial or financial relationships that could be construed as a potential conflict of interest.

## Footnotes

1 www.nationalchamps.com.au
